# Data at your fingertips: How the Integrated Data Service is helping to promote collaboration and efficient decision-making in the UK

**DOI:** 10.23889/ijpds.v8i2.2349

**Published:** 2023-09-14

**Authors:** James Hartley-Binns

**Affiliations:** 1 Office for National Statistics, Titchfield, United Kingdom

The Integrated Data Service (IDS) is a cross-government initiative, designed to transform the way de-identified data is made available for vital research and decision-making about our society and economy.

It will improve the capability for researchers, allowing them to link data effectively through state-of-the-art systems and technologies.

The IDS will enable more collaborative data research and analysis to take place.

The service will enable access to data sets from a range of sources, across government departments and Devolved Administrations, presented alongside analytical and visualisation tools. Linking data to help answer complex policy questions is essential for addressing the cross-cutting and complex issues required to improve today’s society. The Reference Data Management Framework (RDMF) makes it possible to link data on a consistent and convenient basis.

The IDS will link data to provide otherwise untapped insight and to allow rapid analysis on a growing range of integrated data assets. This cross-government service will provide policy makers with robust and collaborative evidence, informing the decisions made across UK society.

This session will introduce IDS to our academic audience, highlighting the benefits for data suppliers, analysts and the wider public that will be available through standardised data, streamlined data linkage, outputs, and the state-of-the-art dissemination technology.

Building on the IDS showcase in April 2023, the presenter will outline the timeline for the continued development of the IDS, and how researchers and existing SRS users can get involved. They will also discuss the ongoing SRS to IDS transition.

The service is open to analysts within the UK government, devolved administrations, and external accredited researchers, such as scientists, academics, and researchers, allowing them to carry out analysis that addresses major societal challenges.

You will learn the advantages of IDS, and the progression the service has made in the last year. The audience will have an increased awareness of the IDS, how to get involved and what the potential impact will be for analysts, users, contributors, and the public.

